# The indirect effects of CMV reactivation on patients following allogeneic hematopoietic stem cell transplantation: an evidence mapping

**DOI:** 10.1007/s00277-023-05509-7

**Published:** 2024-01-16

**Authors:** Xiaojin Wu, Xiao Ma, Tiemei Song, Jie Liu, Yi Sun, Depei Wu

**Affiliations:** 1 The First Affiliated Hospital of Soochow University, Suzhou, 215000 China; 2 National Clinical Research Center for Hematologic Diseases, Jiangsu Institute of Hematology, Suzhou, 215000 China; 3 Institute of Blood and Marrow Transplantation, Collaborative Innovation Center of Hematology, Soochow University, Suzhou, 215000 China; 4 MRL Global Medical Affairs, MSD China, Shanghai, 200233 China

**Keywords:** Cytomegalovirus, Allogeneic hematopoietic stem cell transplantation, Evidence map, Mortality

## Abstract

Cytomegalovirus (CMV) reactivation following allogeneic hematopoietic stem cell transplantation (allo-HSCT) remains a challenging problem, and the impact on the risk of overall mortality (OM) and non-relapse mortality (NRM) in patients following allo-HSCT is still controversial. Utilizing the evidence mapping method, we aimed to assess the effect of CMV infection on outcomes of patients post-transplantation and identify research gaps through systematic reviews (SRs) and clinical studies. PubMed, EMBASE, Web of Science, and Cochrane library databases were searched from inception until 5 July 2022 to identify relevant literature. After systematic literature screening and data extraction, evidence mapping of the effects of CMV reactivation on patients post-allo-HSCT was conducted. Three SRs and 22 clinical studies were included. In one SR, CMV reactivation was associated with an increased risk of mortality (HR 1.46; 95% CI, 1.24–1.72; *P* ≤ 0.001). In two SRs, CMV reactivation was associated with NRM. One SR reported CMV reactivation was potentially associated with significant protection against relapse in patients with acute myelocytic leukemia (AML), but no significant correlation with graft-versus-host disease (GVHD) was found. Lastly, in one SR CMV reactivation significantly increased the risk of invasive fungal disease (IFD). Most clinical articles reported that CMV reactivation increased the risk of renal dysfunction, poor graft function, re-hospitalization, and bacterial infections. CMV reactivation following allo-HSCT is associated with an increased risk of OM, NRM, IFD, and renal dysfunction, as well as a reduced risk of relapse in patients with AML.

**Supplementary Information:**

The online version contains supplementary material available at 10.1007/s00277-023-05509-7.

## Introduction

Allogeneic hematopoietic stem cell transplantation (allo-HSCT) is a potentially lifesaving treatment for patients with hematologic malignancies. However, viral infections remain an important cause of morbidity and mortality following allo-HSCT, especially cytomegalovirus (CMV) reactivation [1], which can occur both early and late post-HSCT [2, 3]. Patients who undergo allo-HSCT are more susceptible to CMV reactivation due to their immunocompromised state. A retrospective study also showed that CMV reactivation was associated with an increased risk of acute graft-versus-host disease (GVHD) in patients who received anti-thymocyte globulin-containing conditioning regimens [4]. Additionally, patients with hematological diseases such as acute myeloid leukemia (AML) and who were post-allo-HSCT are most susceptible to invasive fungal disease (IFD) after CMV reactivation [5]. Patients post-allo-HSCT with CMV seropositivity do not have a better prognosis than patients who have not undergone all-HSCT, despite advances in the diagnosis and management of CMV [3]. However, research findings have been inconsistent [6, 7]. Reactivation [8] of CMV is defined as a new occurrence of CMV antigenemia or CMV DNA in the blood (DNAemia) for patients with CMV-IgG (þ) [9]. Several single-center studies have demonstrated a link between CMV serology/early reactivation of the virus (before 100 days after HCT (D100)) and a reduction in the incidence of relapse of hematological disease [10, 11]. One study of 266 patients with acute myeloid leukemia (AML) who were post-allo-HSCT showed a reduction in the risk of leukemic relapse after early replicative of CMV reactivation, while data from 9469 patients who received a bone marrow or peripheral blood transplantation showed that reactivation of CMV remains a risk factor for poor post-transplant outcomes and does not protect against relapse [3]. In 2019, a meta-analysis of 24 eligible studies with 37,021 patients concluded that while patients with CMV replication who were post-allo-HSCT had a significantly lower risk of relapse, the risk of non-relapse mortality (NRM) was increased [12]. Furthermore, CMV replication was not associated with overall survival (OS) or GVHD disease for patients with AML. Another meta-analysis of 26 studies limited to the English language—17 studies comprising 10, 221 patients evaluated the association between CMV reactivation and the risk of overall mortality (OM), and 14 studies of 18,238 patients assessed the relationship between active CMV reactivation and risk of NRM—reported that patients post-allo-HSCT were at increased risk of OM and NRM after CMV reactivation [13]. However, the increased risk of OM might be offset by an increased risk of NRM. Both meta-analyses had a degree of heterogeneity that could have impacted the reliability of the results. Nonetheless, the above findings suggest controversy still exists about the impact of CMV reactivation on the risk of OM and NRM in patients post-allo-HSCT. Because more evidence has emerged from SRs and primary studies about patient outcomes, this study conducted evidence mapping of the published literature to further investigate the risks associated with CMV reactivation following allo-HSCT.

## Materials and methods

This meta-analysis was performed following the Preferred Reporting Items for Systematic Review and Meta-Analyses (PRISMA) guidelines. This evidence mapping was registered in the International Platform of Registered Systematic Review and Meta-analysis Protocols (INPLASY protocol: 2,022,110,032).

### Data sources and literature search

#### Search strategy and eligibility criteria

Relevant publications were searched by two independent researchers using the PubMed, EMBASE, Web of Science, and Cochrane library databases from inception to 5 July 2022, with the following combination of words, “hematopoietic cell,” “hematopoietic stem,” “transplant,” and “Cytomegalovirus,” limited to the English language. The search strategy used in each database is presented in Online Resource 1. The eligibility criteria were (1) patients undergoing allo-HSCT procedures; (2) patients infected with CMV; (3) systematic reviews (SRs), observational studies, or clinical trials; (4) when two or more studies from the same institution had overlapping populations and assessed the same outcome, the study with the largest number of patients was selected for analysis; (5) studies that reported the correlation between CMV reactivation and primary outcome (OM) or secondary outcomes (NRM, hematologic disease relapse, GVHD, IFD, renal dysfunction, poor graft function, re-hospitalization, and bacterial infections); and (6) because of the level of evidence, the results of systematic reviews were preferentially included and analyzed for each outcome.

### Study selection

Two authors independently screened the titles and abstracts and then reviewed the full texts that met the above-mentioned criteria. Any discrepancies were discussed until a consensus was achieved. Data from published literature was included, while conference abstracts or gray literature was excluded because the data were not peer-reviewed. Furthermore, if outcomes had been reported by SRs, the clinical studies were not searched.

### Data extraction

Data from each study were extracted independently by two separate reviewers. Any disagreements were resolved by discussion with the assistance of a third party if necessary. Baseline characteristics included the type of study, country, number of patients, number of deaths, type of population (allo-HSCT modality), length of follow-up, median age, frequency and duration of CMV monitoring, the method employed for such a purpose (PCR or CMV pp65 antigenemia), type of sample for CMV DNA monitoring, the threshold for initiation of PET, and number of patients with and without CMV reactivation. OM and/or NRM are reported as risk ratio (RR) or hazard ratio (HR) with 95% confidence intervals (CIs).

### Quality assessment

At least two independent investigators applied the Quality In Prognosis Studies (QUIPS) tool to assess the risk of bias in the primary studies. Discrepancies were discussed with another investigator and resolved by discussion. The following 6 domains were included: study participation, study attrition, prognostic factor measurements, outcome measurements, study confounding and statistical analysis, and reporting. The Risk of Bias in Systematic Reviews and the Assessment of Multiple Systematic Reviews (AMSTAR) 2 tool were used to evaluate the risk of bias and quality of included articles.

### Data synthesis and analysis

The frequency or percentage descriptive statistics was used to analyze the data in this study. Excel was utilized to show the methodological quality results of included studies. A summary of interest outcomes was tabulated based on the different outcome measures.

## Results

### Results of study selection

**Fig. 1:**
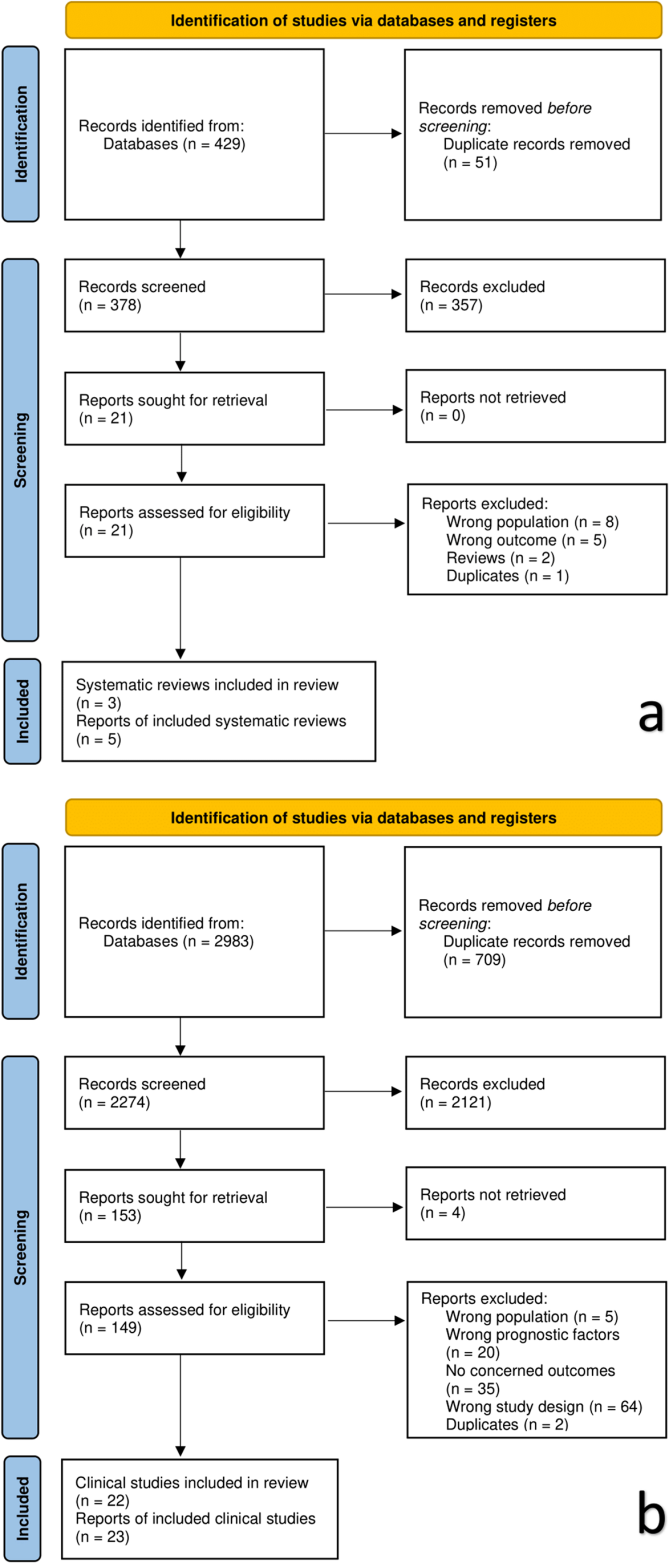
**a** The PRISMA flow chart of systematic review selection. **b** The PRISMA flow chart of clinical study selection

A total of 429 relevant reports were retrieved for SRs, with 3 SRs [12–14] included after the screening. Furthermore, a total of 2983 relevant reports were retrieved for clinical primary studies, and 22 clinical studies [15–36] were ultimately included after screening (Fig. 1a and b).

### Baseline characteristics

The baseline characteristics of the included SRs are presented in Table 1. The 3 SRs were published between 2014 and 2021 (from China, Spanish, and Thailand, respectively), with sample sizes ranging from 7642 to 36,665 cases and which reported the outcomes of OM, NRM, IFD, and disease relapse after allo-HSCT. The baseline characteristics of the included clinical studies are presented in Table 2. The 22 included studies were published between 2005 and 2022 (8 from Asia, 13 from Western countries, and one not available), with sample sizes ranging from 30 to 1825 cases. There were 2 case–control studies and 20 cohort studies that reported the outcomes of renal dysfunction, poor graft function, re-hospitalization, and bacterial infections.

**Table 1:** Characteristics of included systematic reviews

Outcomes	Study ID	No. of original study	Total sample size	Treatment regimen	Prognostic factor	Length of follow-up
All-cause mortality						
	Giménez 2019	16	10,097	NR	CMV infection	≥ 1 year
Non-relapse mortality						
	Giménez 2019	11	8618	NR	CMV infection	≥ 1 year
	Zhang 2019	3	13,274	Patients in 7 of the primary studies received prophylaxis and preemptive antiviral therapy, but no specific drugs were mentioned	CMV replication	≥ 1 year
Hematologic disease relapse						
	Zhang 2019	21	36,665	Patients in 7 of the primary studies received prophylaxis and preemptive antiviral therapy, but no specific drugs were mentioned	CMV replication	≥ 1 year
Graft-versus-host disease						
	Zhang 2019	5	11,745	Patients in 7 of the primary studies received prophylaxis and preemptive antiviral therapy, but no specific drugs were mentioned	CMV replication	≥ 1 year
Invasive fungal disease						
	Chuleerarux 2021	12	7642	Acyclovir; ganciclovir; valganciclovir; phosphonates; immunoglobulins (post-transplant)	CMV reactivation	NR

*CMV*, cytomegalovirus; *NR*, not reported

**Table 2:** Characteristics of included clinical studies

Outcomes	Study ID	Study design	Sample size	Disease type	Transplantation type	Donor type	CMV serostatus	Length of follow-up
Renal dysfunction								
AKI	Madsen 2020	Cohort	408	NR	NR	NR	NR	Median: 23 months
PRF	Deconinck 2005	Cohort	181	AML: 50 (27.6%) ALL: 43 (23.8%) CML: 37 (20.4%) NHL: 26 (14.4%) Myelodysplasia: 15 (8.3%) Myeloma: 3 (1.7%) Hodgkin’s disease: 2 (1.1%) Other: 3 (1.7%)	NR	Identical twins: 2 (1.1%) Related partial match or matched sibling: 149 (82.3%) Unrelated donor: 30 (16.6%)	NR	Median: 52 months (range: 12–128)
Renal impairment	Teschner 2022	Cohort	226	NR	NR	NR	NR	12 months
Poor graft dysfunction								
PGF, primary GR	Chen 2022	Case control study	150	AL: 100 (66.7%) MDS: 31 (20.7%) Lymphoma: 3 (2.0%) MPN: 5 (3.3%) AA: 11 (7.3%)	NR	Matched sibling donor: 85 (56.7%) Non-matched sibling donor: 65 (43.3%)	NR	NR
PGF	Levrat 2016	Cohort	227	NR	NR	HLA-matched donor: 227 (100.0%)	NR	NR
PGF	Prabahran 2021-b	Cohort	819	AML: 299 (36.5%) ALL: 105 (12.8%) CML: 49 (6.0%) CMML: 11 (1.3%) CLL: 44 (5.4%) NHL: 112 (13.7%) HL: 30 (3.7%) MDS: 57 (7.0%) Myelofibrosis: 29 (3.5%) Multiple myeloma: 48 (5.9%) AA: 15 (1.8%) Other: 20 (2.4%)	Bone marrow: 133 (16.3%) Peripheral blood: 685 (83.7%)	Sibling donor: 506 (61.8%) Matched unrelated donor: 299 (36.5%) Haploidentical donor: 8 (1.0%) Matched related: 5 (0.7%)	D−R + /D + R − : 279 (34.1%) D−R − : 204 (24.9%) D + R + : 332 (40.5%) Unknown: 4 (0.4%)	Median: 100 months (95%CI: 89–110)
PGF	Xiao 2014	Cohort	124	ALL: 17 (13.7%) AML: 41 (33.1%) CML: 28 (22.6%) MAL: 3 (2.4%) MDS: 10 (8.1%) NHL: 7 (5.6%) Severe aplastic anemia: 11 (8.9%) Thalassemia: 5 (4.0%)	Bone marrow: 5 (4.1%) Peripheral blood: 89 (73.0%) Bone marrow + peripheral blood: 28 (22.9%)	Unrelated donor: 44 (35.8%) Related donor: 79 (64.2%)	NR	Median: 7 months (range: 1–116 months)
Secondary PGF	Hama 2020	Cohort	49	AA: 49 (100.0%)	Bone marrow: 49 (100.0%)	HLA-matched donor: 29 (59.2%) HLA-mismatched donor: 20 (40.8%)	NR	12 years
Secondary PGF	Sun 2019-a	Cohort	564	AL/MDS: 564 (100.0%)	NR	NR	NR	NR
Secondary PGF	Lv 2021	Cohort	863	AML: 406 (47.0%) ALL: 327 (37.9%) MDS: 89 (10.3%) Others: 41 (4.8%)	NR	Matched sibling donor: 413 (47.9%) Matched unrelated donor: 114 (13.2%) Haploidentical-related donor: 336 (38.9%)	NR	180 days
Secondary PGF	Sun 2019-b	Cohort	490	AML: 231 (47.1%) ALL: 195 (39.8%) MDS: 64 (13.1%)	Bone marrow + peripheral blood: 490 (100%)	Matched sibling donor: 116 (23.7%) Haploidentical donor: 374 (76.3%)	NR	Median: 337 days (range: 71–602)
Secondary PGF	Lin 2022	Cohort	399	AA: 399 (100.0%)	NR	Parent donor: 311 (77.8%) Sibling donor: 65 (16.3%) Offspring donor: 21 (5.4%) Collateral donor: 2 (0.5%)	NR	NR
Re-hospitalization								
Re-hospitalization	Teschner 2022	Cohort	226	NR	NR	NR	NR	12 months
Number of readmission	Miguel 2018	Cohort	170	AML/MDS: 82 (48.2%) ALL: 29 (17.1%) Other: 59 (34.7%)	NR	Matched related donor: 77 (45.3%) Cord blood: 49 (28.8%) Unrelated donor: 36 (21.2%) Haploidentical donor: 8 (4.7%)	D + R + /D + R − /D−R + : 151 (89%)	1 year
Readmission	Prabahran 2021-a	Cohort	30	NR	Bone marrow: 1 (3.3%) Peripheral blood: 29 (96.7%)	Sibling donor: 8 (26.7%) Unrelated donor: 16 (53.3%) Haploidentical donor: 6 (20.0%)	NR	Median: 26 months (95%CI: 23.1–28)
Readmission within 30, 90 days after discharge	Yamagishi 2018	Cohort	156	AML: 76 (48.7%) ALL: 39 (25.0%) MDS: 21 (13.5%) CML: 7 (4.5%) CMML/MPN: 5 (3.2%) NHL/ATL: 7 (4.5%) SAA: 1 (0.6%)	NR	Cord blood: 156 (100.0%)	D + R + /D−R + : 131 (84.0%) D + R−/D−R − : 25 (16.0%)	Median: 82 months (range: 8–160)
Inpatient readmission within 60, 100, 365 days after the index date	Schelfhout 2019-a	Cohort	1825	ALL: 452 (24.8%) AML: 907 (49.7%) CLL: 222 (12.2%) Lymphoma: 590 (32.3%) MDS: 469 (25.7%)	NR	NR	NR	1 year
Bacterial infection								
Nontuberculous mycobacteria infection post-allo-HCT	Beswick 2018	Cohort	1047	AML: 418 (39.9%) ALL: 126 (12.0%) MDS: 104 (9.9%) CML: 95 (9.1%) NHL: 164 (15.7%) AA: 57 (5.4%) Others: 83 (7.9%)	Peripheral blood: 817 (78.0%) Other: 230 (22.0%)	Related donor: 541 (53.1%) Unrelated donor: 477 (46.9%)	NR	Median: 51 months (IQR: 23–70)
Clostridium difficile infection	Lavallée 2016	Case control study	188	AML: 49 (26.1%) ALL: 26 (13.8%) CML: 16 (8.5%) CLL: 6 (3.2%) MM: 16 (8.5%) Lymphoma: 45 (23.9%) MDS: 19 (10.1%) Others: 11 (5.9%)	Bone marrow: 36 (19.1%) Cord blood: 5 (2.7%) Peripheral blood: 147 (78.2%)	Matched related donor: 112 (59.6%) Mismatched or unrelated donor: 76 (40.4%)	NR	NR
Blood stream infection	Sano 2017	Cohort	278	NR	NR	Matched related donor: 112 (40.3%) Mismatched related donor: 19 (6.8%) Unrelated donor: 71 (25.5%) Cord blood: 76 (27.3%)	D + R + : 50 (18.0%) D + R−: 28 (10.1%) D−R + : 102 (36.7%) D−R−: 98 (35.3%)	10 years
Any bacteremia	Saullo 2020	Cohort	388	AL: 183 (47.2%) Lymphoma: 81 (20.9%) MDS/MPN: 61 (15.7%) Others: 63 (16.2%)	Bone marrow: 27 (7.0%) Cord blood: 66 (17.0%) Peripheral blood: 295 (76.0%)	Matched unrelated donor: 175 (45.1%) Matched related donor: 107 (27.6%) Mismatched unrelated donor: 71 (18.3%) Mismatched related donor: 35 (9.0%)	D + R + : 109 (28.1%) D + R − : 57 (14.7%) D−R + : 106 (27.3%) D−R − : 91 (23.5%) Indeterminate/unknown: 25 (6.4%)	NR
Bacterial infection	Skert 2014	Cohort	35	AL: 16 (45.7%) NHL/CLL: 8 (22.9%) Other: 11 (31.4%)	Bone marrow: 4 (11.4%) Peripheral blood: 31 (88.6%)	Matched related donor: 23 (65.7%) Matched unrelated donor: 12 (34.3%)	NR	120 days
Bacterial infection	Vinuesa 2016	Cohort	170	AML: 64 (37.7%) NHL: 41 (24.1%) MDS: 8 (4.7%) ALL: 14 (8.2%) CLL: 13 (7.7%) MM: 6 (3.5%) Hodgkin’s lymphoma: 8 (4.7%) AA: 1 (0.6%) Others: 15 (8.8%)	Bone marrow: 7 (4.1%) Cord blood: 35 (20.2%) Peripheral blood: 131 (75.7%)	HLA-matched donor: 132 (76.3%) HLA-mismatched donor: 41 (23.7%)	D + R + : 99 (57.2%) D + R − : 10 (5.8%) D−R + : 64 (37.0%)	60 days

*AA*, aplastic anemia; *AKI*, acute kidney injury; *AML*, acute myeloblastic leukemia; *ALL*, acute lymphoblastic leukemia; *ATL*, adult T cell leukemia–lymphoma; *CMV*, cytomegalovirus; *CLL*, chronic lymphoblastic leukemia; *CML*, chronic myeloid leukemia; *CMML*, chronic myelomonocytic leukemia; *D*, donor; *GR*, graft rejection; *HL*, Hodgkin’s lymphoma; *HLA*, human leukocyte antigen; *IQR*, interquartile range; *MAL*, mixed lineage acute leukemia; *MDS*, myelodysplastic syndrome; *MM*, multiple myeloma; *MPN*, myeloproliferative neoplasm; *NHL*, non-Hodgkin’s lymphoma; *NR*, not reported; *PGF*, primary poor graft function; *PRF*, prolonged renal failure; *R*, recipient; *SAA*, severe aplastic anemia

### The results of the quality of the included studies

Results of the AMSTAR 2 assessment are shown in Online Resource 2. For each AMSTAR 2 item, among the 16 items, nine items were rated as “Yes” (items 1, 3, 5, 6, 11, 13, 14, 15, and 16) for Giménez 2019 [13], nine items were rated as “Yes” (items 1, 3, 5, 9, 11, 13, 14, 15, and 16) for Chuleerarux 2021 [14], eight items were rated as “Yes” (items 1, 5, 6, 9, 11, 14, 15, and 16) for Zhang 2019 [12]. In addition, included primary clinical studies whose overall risk bias was all assessed with “Moderate” are shown in Online Resource 3.

### Primary outcome

#### All-cause mortality

**Fig. 2:**
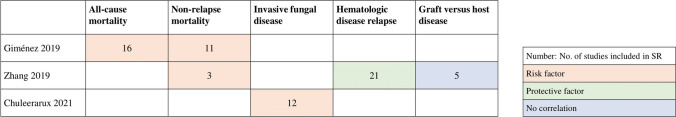
Summary of evidence from systematic reviews

One SR [13] included 16 studies comprising 10,097 patients published in 2019 reported the outcomes of all-cause mortality and showed that CMV reactivation was associated with an increased risk of OM (HR 1.46; 95% CI, 1.24–1.72; *P* ≤ 0.001) (Table 3, Fig. 2).

**Table 3:** Summary of evidence from systematic reviews

Outcomes	Study ID	Prognostic factor	Conclusion	Significant correlation
All-cause mortality				
	Giménez 2019	CMV infection	CMV infection was associated with an increased risk of overall mortality (HR 1.46; 95% CI, 1.24–1.72; *P* ≤ 0.001)	Yes
Non-relapse mortality				
	Giménez 2019	CMV infection	CMV infection was associated with an increased risk of non-relapse mortality (HR 1.41; 95% CI, 1.08–1.83; *P* ≤ 0.01)	Yes
	Zhang 2019	CMV replication	CMV replication was associated with an increased risk of non-relapse mortality for AML (HR 1.64; 95% CI, 1.46–1.85; *P* ≤ 0.001) and ALL (HR 1.92; 95% CI, 1.57–2.34; *P* ≤ 0.001)	Yes
Hematologic disease relapse				
	Zhang 2019	CMV replication	CMV replication was a significant protection against disease relapse (HR 0.74; 95% CI, 0.63–0.87; *P* < 0.001). CMV replication was associated with a decreased risk of relapse for AML (HR 0.64; 95% CI, 0.50–0.83; *P* < 0.001) but not for ALL	Yes
Graft-versus-host disease				
	Zhang 2019	CMV replication	CMV replication was not associated with GVHD for AML (acute GVHD: HR 0.87; 95% CI, 0.55–1.39; *P* = 0.564; chronic GVHD: HR 0.88; 95% CI, 0.38–2.03; *P* = 0.758) and ALL (acute GVHD: HR 1.24; 95% CI, 0.98–1.57; *P* = 0.078)	No
Invasive fungal disease				
	Chuleerarux 2021	CMV reactivation	Post-transplant CMV significantly increased the risk of subsequent IFDs (HR 2.575; 95% CI, 1.775–3.737; *P* < 0.001)	Yes

*AML*, acute myeloblastic leukemia; *ALL*, acute lymphoblastic leukemia; *CMV*, cytomegalovirus; *GVHD*, graft-versus-host disease; *HR*, hazard ratio; *IFD*, invasive fungal disease

### Secondary outcome

#### Non-relapse mortality (NRM)

Two SRs reported the outcomes of non-relapse mortality. One study [13] included 11 studies comprising 8618 patients published in 2019 and reported that CMV reactivation was associated with NRM (HR 1.41; 95% CI, 1.08–1.83; *P* = 0.01). Another study [12] included three studies consisting of 13,274 patients published in 2019 and reported that CMV replication was an independent risk factor for increased non-relapse mortality for AML (HR 1.64; 95% CI, 1.46–1.85; *P* < 0.001) and ALL (HR 1.92; 95% CI, 1.57–2.34; *P* < 0.001) (Table 3, Fig. 2).

#### Hematologic disease relapse

One SR [12] included 21 studies that reported the outcome of hematologic disease relapse. The meta-analysis published in 2019 included 36,665 patients and reported there might be a significant correlation between disease relapse and CMV replication (HR 0.74; 95% CI, 0.63–0.87; *P* < 0.001). And there was a significant protection against relapse observed in the AML patients (HR 0.64; 95% CI, 0.50–0.83; *P* < 0.001) (Table 3, Fig. 2).

#### Graft-versus-host disease (GVHD)

One SR [12] included 5 studies with 11,745 patients and evaluated the relationship between CMV reactivation and GVHD. There was no association between CMV replication and GVHD for AML-aGVHD (HR 0.87 (0.55–1.39; *P* = 0.564), AML-cGVHD (HR 0.88 (0.38–2.03); *P* = 0.758), and ALL-aGVHD (HR 1.24 (0.98–1.57); *P* = 0.078) (Table 3, Fig. 2).

#### Invasive fungal disease (IFD)

One SR [14] published in 2021 included 12 studies with 7642 patients and evaluated the relationship between CMV reactivation and IFD. CMV reactivation significantly increased the risk of IFD (HR 2.575; 95% CI, 1.775–3.737; *P* < 0.001) (Table 3, Fig. 2).

#### Renal dysfunction, poor graft function, re-hospitalization, and bacterial infections

**Fig. 3:**
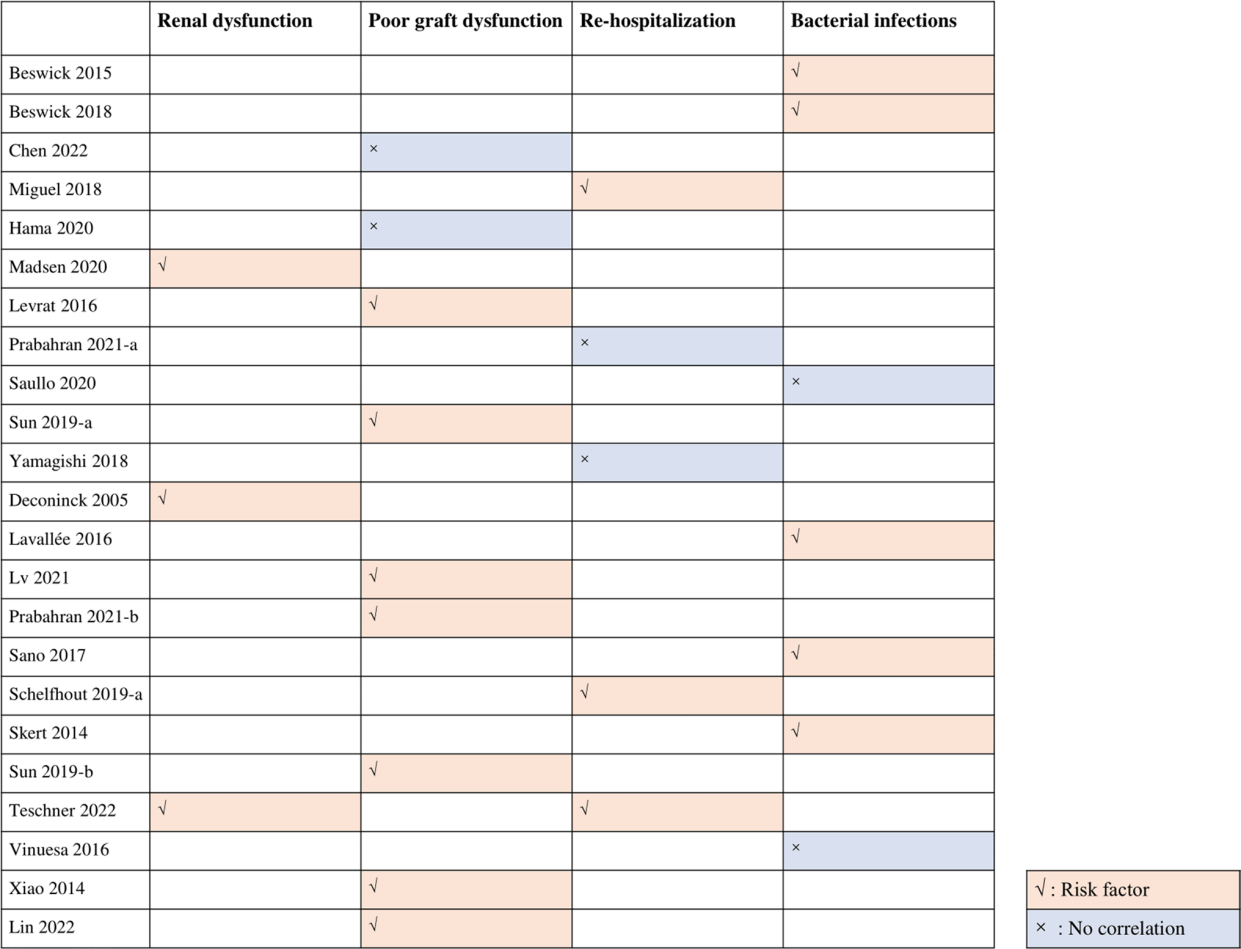
Summary of evidence from primary clinical studies

Three studies [18, 23, 33] included 815 patients and support that CMV reactivation increases the risk of renal dysfunction in patients after allo-HSCT (Table 4, Fig. 3). Seven studies [21, 22, 24, 25, 31, 32, 35] support that CMV reactivation increases the risk of poor graft function, while two studies [16, 19] showed no association between CMV reactivation and poor graft function (Table 4, Fig. 3). Three studies [17, 29, 33] support that CMV reactivation increases the risk of re-hospitalization, but two studies [25, 36] found no association between CMV reactivation and re-hospitalization (Table 4, Fig. 3). Four of six studies [15, 20, 27, 28, 30, 34] support that CMV reactivation increases the risk of bacterial infections (Table 4, Fig. 3).

**Table 4:** Summary of evidence from primary clinical studies

Outcomes	Study ID	Prognostic factor	Conclusion	Significant correlation
Renal dysfunction				
AKI	Madsen 2020	CMV reactivation	More than two CMV reactivations was associated with post-transplant AKI (*P* = 0.02)	Yes
PRF	Deconinck 2005	CMV infection	Multivariate analysis: CMV infection was a significant impact on post-transplant PRF (OR 3.29; 95% CI, 1.13–9.59; *P* = 0.01)	Yes
Renal impairment	Teschner 2022	CMV infection or disease	Renal impairment: CMV group (*n* = 66): 44 (66.7%), no CMV group (*n* = 160): 71 (44.4%), *P* = 0.003	Yes
Poor graft dysfunction				
PGF, primary GR	Chen 2022	CMV infection	Univariate analysis: PGF: CMV group (*n* = 2): 1 (50.0%), no CMV group (*n* = 93): 18 (19.4%), *P* = 0.858	No
PGF	Levrat 2016	CMV infection	CMV negative donor/recipient pair was associated lower risk of PGF (*P* = 0.04)	Yes
PGF	Prabahran 2021-b	CMV viremia	Multivariate analysis: CMV viremia was significantly associated with development of PGF (OR 2.43; 95% CI, 1.53–3.88; *P* < 0.001)	Yes
PGF	Xiao 2014	CMV infection or disease	Multivariate analysis: CMV infection in 30 days was significantly associated with PGF (OR 9.146; 95% CI, 1.153–55.276; *P* = 0.016)	Yes
Secondary PGF	Hama 2020	CMV reactivation	Multivariate analysis: CMV reactivation was not significantly associated with PGF (HR 1.68; 95% CI, 0.42–6.63; *P* = 0.46)	No
Secondary PGF	Sun 2019-a	CMV reactivation	CMV reactivation was significantly associated with secondary PGF (HR 7.827; 95% CI, 2.002–30.602; *P* = 0.003)	Yes
Secondary PGF	Lv 2021	CMV reactivation	Multivariable analysis: CMV reactivation was identified as independent hazard elements of secondary PGF (HR 8.915; 95% CI, 5.100–15.985; *P* < 0.0001)	Yes
Secondary PGF	Sun 2019-b	CMV reactivation	Multivariable analysis: CMV reactivation was identified as independent risk factors of secondary PGF (HR 7.827; 95% CI, 2.002–30.602; *P* = 0.003)	Yes
Secondary PGF	Lin 2022	CMV reactivation	Multivariate analysis: CMV reactivation was significantly associated with PGF (Model 1: OR 6.020; 95% CI, 1.664–21.785; *P* = 0.006; model 2: OR 6.986; 95% CI, 2.002–24.379; *P* = 0.002)	Yes
Re-hospitalization				
Re-hospitalization	Teschner 2022	CMV infection or disease	The re-hospitalization rate was significantly higher among patients with CMV during the follow-up period compared to those without a respective diagnosis (90.7 vs. 76.3%, *P* = 0.004)	Yes
Number of readmission	Miguel 2018	CMV reactivation	Patients with CMV reactivation had an increased number of readmissions during the first year after HCT (2 ± 1.6 versus 1.3 ± 1.4; *P* = 0.048)	Yes
Readmission	Prabahran 2021-a	CMV reactivation	CMV reactivation and CMV therapy contributed to 13/17 (76%) total readmissions for those with recurrent CMV/prolonged persisting infection compared to 5/13 (38%) readmissions for those with single CMV reactivation	No
Readmission within 30, 90 days after discharge	Yamagishi 2018	Recipient CMV serostatus	Multivariate analysis: recipient CMV serostatus was not significantly associated with readmission after discharge (30 days: HR 0.34; 95% CI, 0.06–1.69; *P* = 0.188; 90 days: HR 0.44; 95% CI, 0.14–1.40; *P* = 0.166)	No
Inpatient readmission within 60, 100, 365 days after the index date	Schelfhout 2019-a	CMV infection or disease	Patients with CMV infection were significantly more likely to have a 60-day (31.2% vs. 19.4%), 100-day (50.0% vs 30.5%) or 365-day readmission (78.0% vs 57.8%) compared to those without a CMV-related event (all *P* < 0.001)	Yes
Bacterial infection				
Nontuberculous mycobacteria infection post-allo-HCT	Beswick 2018	CMV viremia	Multivariate analysis: CMV viremia was significantly associated with nontuberculous mycobacteria infection post-allo-HCT (HR 5.77; 95% CI, 1.71–19.45; *P* = 0.004)	Yes
* Clostridium difficile* infection	Lavallée 2016	CMV reactivation	Multivariate analysis: CMV reactivation was significantly associated with increased risk of *Clostridium difficile* infection (OR 6.17; 95% CI, 2.17–17.57; *P* = 0.001)	Yes
Blood stream infection	Sano 2017	CMV viremia	Multivariate analysis: CMV viremia was significantly associated with risk for blood stream infections (HR 3.34; 95% CI, 1.51–7.36; *P* = 0.003)	Yes
Any bacteremia	Saullo 2020	CMV infection	Clinically significant CMV infection was not significantly associated with incidence of bacteremia: 59 (34.7%) vs. 65 (29.8%), *P* = 0.202	No
Bacterial infection	Skert 2014	CMV infection	Multivariate analysis: CMV infection before day 30 showed a positive correlation with bacterial infections (HR 50; 95% CI, 7–54; *P* = 0.003)	Yes
Bacterial infection	Vinuesa 2016	CMV DNAemia	Univariate analysis: the occurrence of CMV DNAemia was not found to be a risk factor for bacterial infection (HR 1.00; 95% CI, 0.57–1.75; *P* = 0.993)	No

*AKI*, acute kidney injury; *CMV*, cytomegalovirus; *DNA*, deoxyribonucleic acid; *GR*, graft rejection; *GVHD*, graft-versus-host disease; *HCT*, hematopoietic stem cell transplantation; *HR*, hazard ratio; *OR*, odds ratio; *PGF*, poor graft function; *PRF*, prolonged renal failure

## Discussion

The effect of CMV reactivation post-allo-HSCT, especially the indirect effect, is multifaceted, and the related studies are intricate. This is the first evidence mapping study in this field that provides a more comprehensive assessment of the progress made and the current status of the field. The results suggest that CMV reactivation is associated with an increased risk of OM and NRM for patients after allo-HSCT. Furthermore, CMV reactivation might be associated with an increased risk of IFD, renal dysfunction, poor graft function, bacterial infections, and re-hospitalization. Finally, CMV reactivation might be protective against hematologic disease relapse.

CMV reactivation is one of the most common causes of morbidity and mortality following allo-HSCT and occurs primarily within the first 100 days post-transplantation. Despite advances in the diagnosis and prevention of CMV reactivation, previous studies have reported a significantly lower median survival rate and increased overall mortality in patients with reactivation following transplantation [37–40]. One SR included 17 studies comprising 10,221 patients showed CMV reactivation was significantly associated with an increased risk of OM. Furthermore, the use of preemptive antiviral therapy led to a twofold increase risk in the risk of OM [13]. Thus, based on the available evidence, CMV reactivation is associated with an increased risk of OM. Most studies found a significant correlation between CMV reactivation after transplantation and an increase in NRM [41, 42]. Two SRs included in the systematic review provided sufficient data to support that CMV reactivation was significantly associated with an increased risk of NRM [12, 13].

Several recent studies have reported that CMV reactivation following allo-HCT reduced the risk of early relapse in patients with AML but was not associated with a reduced risk in patients with other diseases [42]. Results, however, have been inconsistent. One study from the Center for International Blood and Marrow Transplant Research (CIBMTR) Database showed that CMV reactivation after allo-HCT was not associated with relapse in patients with AML [3]. Furthermore, the results from European Conference on Infections in Leukemia (ECIL) [43] and American Society of Transplantation and Cellular Therapy (ASTCT)[44] also found no association between CMV reactivation and relapse in patients after allo-HCT. The SR published in 2019 showed a significantly lower relapse risk after allo-HSCT in patients with AML and CMV replication [12]. The above studies suggest that the correlation between CMV reactivation and tumor recurrence is still highly controversial. Furthermore, the mechanisms underlying the reduction in the rate of relapse are unclear. Repeated environmental influences such as CMV have profound effects on immune homeostasis and the immune system in general, especially on T cells that are involved in anti-tumor immunity [45]. One recent study reported that aside from CMV-CTL reconstitution, CMV reactivation could affect WT1-specific CD8 + T cell reconstitution following allo-HSCT, potentially contributing to the remission or relapse of AML. Moreover, although CMV-CTL reconstitution may be beneficial in reducing CMV activation, it may be detrimental to immune reconstitution. CMV-CTL reconstitution is often accompanied by a reduction in naive T cells and a stronger immune response, both of which also reflect the possible correlation between CMV and GVHD and the increased risk of subsequent opportunistic infections [46]. Patients with both aGVHD and CMV reactivation had significantly higher NRM and poorer OS [10, 47]. Because infections with CMV and GVHD are the most common complications and account for most of the deaths following allo-HSCT, it is necessary to clarify the relationship between GVHD and CMV reactivation after allo-HSCT. Although the SR showed no association between CMV replication and GVHD [12], one study consisting of 515 patients who underwent allo-HSCT between 1993 and 2008 showed that during phases of CMV replication, patients were at increased risk of developing acute GVHD [48].

IFD is another important infectious complication that cannot be ignored. IFD may be caused by CMV reactivation or side effects of antiviral drugs. Although several previous studies demonstrated that CMV reactivation was a risk factor for IFD, other studies reported conflicting results [5, 49–51]. Other factors that must be considered are transplant-related factors, use of corticosteroids, neutropenia induced by anti-CMV drugs such as ganciclovir, and/or the adverse effects on host immunity by CMV itself [51]. Additionally, most studies included in the evidence mapping research support that CMV reactivation is associated with an increased risk of renal dysfunction, poor graft function, and bacterial infections in allo-HSCT recipients. Foscarnet, which is used to treat ganciclovir-resistant CMV infections, is efficacious but also associated with nephrotoxicity, with rates as high as 60% during therapy due primarily to acute tubulointerstitial nephritis, which can lead to renal dysfunction [52]. Furthermore, side effects of drugs given for complications of CMV, for example, aminoglycosides given for neutropenic fever due to poor graft function, can lead to severely reduced kidney function [53]. Poor graft function is a life-threatening complication following allo-HSCT. Prabahran and his colleagues [25] demonstrated that CMV viremia [OR 2.43; 95% CI, 1.53–3.88; *P* < 0.001] was significantly associated with the development of poor graft function. CMV infection in patients following allo-HSCT has been shown to decrease the expression of bone stroma secretion factors and lead to poor graft function [54]. Other reasons for poor graft function included the use of ganciclovir, number of infused CD34 + cells, presence of HLA antibodies, and GVHD [55].

Infection is one of the most common complications in patients with allo-HSCT, with the most common infection being bacteremia, especially gram-negative bacteremia [56]. Approximately one-fifth of patients post-HSCT develop bacteremia concurrently with CMV reactivation [57]. While risk factors for bacterial infection vary, levofloxacin prophylaxis in HSCT recipients is associated with the emergence of fluoroquinolone-resistant gram-negative bacteria [58]. Recently, valganciclovir and ganciclovir have been successfully used for the prevention and treatment of CMV reactivation, although it is associated with serious side effects such as leukopenia, which can lead to bacterial infections [59]. One randomized controlled trial of 565 patients who received either letermovir or placebo from 2014 to 2016 found that prophylactic letermovir treatment significantly reduced the risk of clinically significant CMV reactivation compared to placebo [60]. A previous study demonstrated through week 24 post-transplantation, all-cause mortality rate was 15.0% in the letermovir group versus 18.2% in the placebo group; with rates of 26.5% and 40.9%, respectively, through week 48 [61].

## Conclusion

The impact of CMV reactivation post-allo-HSCT is substantial and is associated with an increased risk of OM, NRM, IFD, hematologic disease relapse, renal dysfunction, re-hospitalization, poor graft function, and bacterial infections. A proactive and adequate course of therapy to prevent CMV is necessary. Further attention needs to be paid to the value of using letermovir for CMV prophylaxis and to improving the prognosis of patients with CMV reactivation post-allo-HSCT in the future.

### Supplementary Information

Below is the link to the electronic supplementary material.

Supplementary file1 (PDF 170 KB)

Supplementary file2 (PDF 164 KB)

Supplementary file3 (PDF 167 KB)

## Data Availability

The original contributions presented in this study are included in the article/Online Resource material. Further inquiries can be directed to the corresponding author.
