# Supplementary material for: The indirect effects of CMV reactivation on patients following allogeneic hematopoietic stem cell transplantation: an evidence mapping
Source: Ann Hematol. 2024 Jan 16;103(3):917–33. doi: 10.1007/s00277-023-05509-7 (PMC10866798; doi:10.1007/s00277-023-05509-7)
Supplement: Supplementary file 1 — Supplementary file1 (PDF 170 KB) [file 277_2023_5509_MOESM1_ESM.pdf]

*Annals of Hematology*

**The Indirect Effects of CMV Infection on Patients with Allogeneic Hematopoietic Stem Cell transplantation: an Evidence Mapping**

Xiaojin Wu[1,2,3], Xiao Ma[1,2], Tiemei Song[1,2], Jie Liu[4], Yi Sun [4], Depei Wu[1,2*]

1. The First Affiliated Hospital of Soochow University, Suzhou, 215000, China

2. National Clinical Research Center for Hematologic Diseases, Jiangsu Institute of Hematology, Suzhou, 215000, China.

3. Institute of Blood and Marrow Transplantation, Collaborative Innovation Center of Hematology, Soochow University, Suzhou, 215000, China.

4. MRL Global Medical Affairs, MSD China, Shanghai, 200233, China.

**Corresponding author:**

Depei Wu, National Clinical Research Center for Hematologic Diseases, Jiangsu Institute of Hematology, The First Affiliated Hospital of Soochow University, 188 Shizi Street, Suzhou, Jiangsu Province 215006, China. Email: drwudepei@163.com .

# Online Resource 1 Literature search strategy

## Systematic review

| Database | Search Strategy |
|---|---|
| PubMed | #1 "Hematopoietic Stem Cell Transplantation"[MeSH Terms]<br>#2 ("hematopoietic cell*"[tiab] or "hematopoietic stem*"[tiab] ) and Transplant*[tiab] or "HSCT"[tiab] or "HCT"[tiab]<br>#3 "Cytomegalovirus Infections"[Mesh]<br>#4 "Cytomegalovirus"[Mesh]<br>#5 "Cytomegalovir*"[tiab] OR "CMV"[tiab]<br>#6 (Systematic Review[Publication Type]) OR (meta analysis[Publication Type])<br>#7 "systematic review"[tiab] OR "meta"[tiab]<br>#8 (#1 OR #2) and (#3 OR #4 OR #5) and (#6 OR #7) |
| EMBASE | #1 'hematopoietic stem cell transplantation'/exp<br>#2 ('hematopoietic cell*' OR 'hematopoietic stem*') AND transplant* OR 'hsct' OR 'hct'<br>#3 'cytomegalovirus infection'/exp<br>#4 'cytomegalovirus'/exp<br>#5 "Cytomegalovir*" OR "CMV"<br>#6 "Systematic Review" or "meta"<br>#7 (#1 OR #2) and (#3 OR #4 OR #5) AND #6 |
| Cochrane Library | #1 MeSH descriptor: [Hematopoietic Stem Cell Transplantation] explode all trees<br>#2 ('hematopoietic cell*' OR 'hematopoietic stem*') AND transplant* OR 'hsct' OR 'hct'<br>#3 MeSH descriptor: [Cytomegalovirus Infections] explode all trees<br>#4 MeSH descriptor: [Cytomegalovirus] explode all trees<br>#5 "Cytomegalovir*" OR "CMV"<br>#6 #1 OR #2<br>#7 #3 OR #4 OR #5<br>#8 #6 AND #7 |
| Web of Science | ( ('hematopoietic cell*' OR 'hematopoietic stem*') AND transplant* OR 'hsct' OR 'hct' )<br>And Cytomegalovir* and ("Systematic Review" or "meta") |

## Clinical study

| Database | Search Strategy |
|---|---|
| PubMed | #1. "Cytomegalovirus"[Mesh] OR "Cytomegalovirus Infections"[Mesh] OR Cytomegalovirus*[tw] OR "Salivary Gland Virus*"[tw] OR "Human Herpesvirus 5"[tw] OR "HHV 5"[tw] OR Cytomegalic[tw] OR CMV[tw] OR HCMV[tw] OR Cytomegaly[tw] OR cytomegalia[tw] OR cytomegalo[tw] OR cytomegalus[tw] OR cytomegalusvirus*[tw] OR "human herpes virus 5"[tw] OR cytomegaloinfection*[tw] OR cytomegaloviral[tw]<br>#2. "Allografts"[Mesh] OR Allogeneic[tw] OR allogenic[tw] OR allograft[tw] OR allotransplantation*[tw] OR "Allo Transplantation*"[tw] OR Homologous[tw] OR Homograft*[tw]<br>#3. "Stem Cell Transplantation"[Mesh] OR "Bone Marrow Transplantation"[Mesh] OR "Bone Marrow Grafting"[tw] OR "Bone Marrow Cell Transplantation*"[tw] OR "Bone Marrow Transplantation*"[tw] OR "stem cell transplantation*"[tw] OR "hematopoietic cell transplantation*"[tw] OR "stem cell Grafting"[tw]<br>#4. "Haploidentical Transplantation*"[tw] OR "unrelated donor transplantation*"[tw] OR "Cord Blood Stem Cell Transplantation"[Mesh] OR "Cord Blood Transplantation*"[tw] OR "Umbilical cord blood Transplantation*"[tw] OR "Umbilical blood Transplantation*"[tw] OR "Semicongruent Transplantation*"[tw] OR Halfmatched[tw] OR "Half matched"[tw] OR "allo-HSCT"[tw]<br>#5. #1 AND ((#2 AND #3) OR #4)<br>#6. "Neutropenia"[Mesh] OR Neutropenia*[tw] OR neutropenic[tw] OR neutropaenia*[tw] OR "Barth syndrome"[tw] OR "Evans syndrome"[tw]<br>#7. "Renal Insufficiency"[Mesh] OR Renal Insufficienc*[tw] OR Kidney Insufficienc*[tw] OR Kidney Failure*[tw] OR Renal Failure*[tw] OR Renal dysfunction*[tw] OR Kidney dysfunction*[tw] OR "Acute Kidney Injur*"[tw] OR "Acute Renal Injur*"[tw] OR "Lower Nephron Nephrosis"[tw] OR "Lower Nephron Nephroses"[tw] OR "Kidney Tubular Necrosis"[tw] OR "Kidney Tubular Necroses"[tw] OR "Cardio Renal Syndrome*"[tw] OR "Reno-Cardiac Syndrome*"[tw] OR "Renocardiac Syndrome*"[tw] OR "Cardiorenal Syndrome*"[tw] OR "Chronic Kidney Disease*"[tw] OR "Chronic Renal Disease*"[tw] OR "Frasier Syndrome*"[tw] |

| | |
|---|---|
| | #8. "Primary Graft Dysfunction"[Mesh] OR graft dysfunction*[tw] OR "Poor graft dysfunction*"[tw] OR "Poor graft function*"[tw] OR PGF[tw] OR IPGF[tw]<br>#9. "Re-hospital*"[tw] OR readmis*[tw] OR "re-admit*"[tw] OR readmit*[tw] OR "re-admis*"[tw]<br>#10. "Bacterial Infections"[Mesh] OR bacteri*[tw] OR Actinomycetal*[tw] OR Corynebacteri*[tw] OR diphtheria[tw] OR actinomycos*[tw] OR mycobacterios*[tw] OR leprosy[tw] OR paratuberculos*[tw] OR tuberculo*[tw] OR nocardios*[tw] OR anthrax[tw] OR melioidos*[tw] OR Bacteremia[tw] OR Septicemia[tw] OR Anaplasmata*[tw] OR Anaplasmos*[tw] OR Bartonella*[tw] OR Bordetella[tw] OR Brucellos*[tw] OR Burkholderia*[tw] OR Campylobact*[tw] OR Chlamydiac*[tw] OR Cytophagac*[tw] OR Desulfovibrionac*[tw] OR Ehrlichios*[tw] OR Enterobacteriac*[tw] OR Flavobacteriac*[tw] OR Fusobacteriac*[tw] OR Helicobacter*[tw] OR Legionellos*[tw] OR Moraxellac*[tw] OR Mycoplasmata*[tw] OR Neisseriac*[tw] OR Pasteurellac*[tw] OR Piscirickettsiac*[tw] OR Pseudomon*[tw] OR Rickettsiac*[tw] OR Spirochaetal*[tw] OR Treponema*[tw] OR Tularemia[tw] OR Vibrio[tw] OR Actinomycet*[tw] OR Bacillac*[tw] OR Clostridi*[tw] OR Erysipelothr*[tw] OR Listerios*[tw] OR Staphylococc*[tw] OR Streptococc*[tw] OR Chancroid[tw] OR Chlamydia[tw] OR Vaginos*[tw]<br>#11. #5 AND (#6 OR #7 OR #8 OR #9 OR #10) |
| EMBASE | #1. 'Cytomegalovirus'/exp OR 'cytomegalovirus infection'/exp OR (Cytomegalovirus* OR "Salivary Gland Virus*" OR "Human Herpesvirus 5" OR "HHV 5" OR Cytomegalic OR CMV OR HCMV OR Cytomegaly OR cytomegalia OR cytomegalo OR cytomegalus OR cytomegalusvirus* OR "human herpes virus 5" OR cytomegaloinfection* OR cytomegaloviral):ab,ti,kw<br>#2. 'allogeneic stem cell transplantation'/exp OR 'allogenic bone marrow transplantation'/exp OR 'cord blood stem cell transplantation'/exp OR (((Allogeneic OR allogenic OR allograft OR allotransplantation* OR Homologous OR Homograft* OR Allo) NEAR/4 ("Bone Marrow" OR "stem cell" OR "hematopoietic cell") NEAR/4 (Grafting OR Transplantation*)) OR ((Haploidentical OR "unrelated donor" OR "Cord Blood" OR "Umbilical cord blood " OR "Umbilical blood" OR Semicongruent) NEAR/3 (Grafting OR Transplantation*)) OR Halfmatched OR "Half matched" OR "allo-HSCT"):ab,ti,kw<br>#3. #1 AND #2<br>#4. 'neutropenia'/exp OR (Neutropenia* OR neutropenic OR neutropaenia* OR "Barth syndrome" OR "Evans syndrome"):ab,ti,kw<br>#5. 'kidney failure'/exp OR (((Renal OR Kidney) NEAR/3 (Insufficienc* OR Failure* OR |

| | |
|---|---|
| | dysfunction*)) OR "Acute Kidney Injur*" OR "Acute Renal Injur*" OR "Lower Nephron Nephrosis" OR "Lower Nephron Nephroses" OR "Kidney Tubular Necrosis" OR "Kidney Tubular Necroses" OR "Cardio Renal Syndrome*" OR "Reno-Cardiac Syndrome*" OR "Renocardiac Syndrome*" OR "Cardiorenal Syndrome*" OR "Chronic Kidney Disease*" OR "Chronic Renal Disease*" OR "Frasier Syndrome*"):ab,ti,kw<br>#6. 'graft dysfunction'/de OR ("graft dysfunction*" OR "Poor graft function*" OR PGF OR IPGF):ab,ti,kw<br>#7. ("Re-hospital*" OR readmis* OR "re-admit*" OR readmit* OR "re-admis*"):ab,ti,kw<br>#8. 'bacterial infection'/exp OR (bacteri* OR Actinomycetal* OR Corynebacteri* OR diphtheria OR actinomycos* OR mycobacterios* OR leprosy OR paratuberculos* OR tuberculo* OR nocardios* OR anthrax OR melioidos* OR Bacteremia OR Septicemia OR ((Anaplasmata* OR Anaplasmos* OR Bartonella* OR Bordetella OR Brucellos* OR Burkholderia* OR Campylobact* OR Chlamydiac* OR Cytophagac* OR Desulfovibrionac* OR Ehrlichios* OR Enterobacteriac* OR Flavobacteriac* OR Fusobacteriac* OR Helicobacter* OR Legionellos* OR Moraxellac* OR Mycoplasmata* OR Neisseriac* OR Pasteurellac* OR Piscirickettsiac* OR Pseudomon* OR Rickettsiac* OR Spirochaetal* OR Treponema* OR Tularemia OR Vibrio OR Actinomycet* OR Bacillac* OR Clostridi* OR Erysipelothr* OR Listerios* OR Staphylococc* OR Streptococc* OR Chancroid OR Chlamydia OR Vaginos*) NEAR/4 infect*)):ab,ti,kw<br>#9. #3 AND (#4 OR #5 OR #6 OR #7 OR #8) |
| Cochrane Library | #1 (Cytomegalovirus* OR "Salivary Gland Virus*" OR "Human Herpesvirus 5" OR "HHV 5" OR Cytomegalic OR CMV OR HCMV OR Cytomegaly OR cytomegalia OR cytomegalo OR cytomegalus OR cytomegalusvirus* OR "human herpes virus 5" OR cytomegaloinfection* OR cytomegaloviral):ti,ab,kw<br>#2 (((Allogeneic OR allogenic OR allograft OR allotransplantation* OR Homologous OR Homograft* OR Allo) NEAR/4 ("Bone Marrow" OR "stem cell" OR "hematopoietic cell") NEAR/4 (Grafting OR Transplantation*)) OR ((Haploidentical OR "unrelated donor" OR "Cord Blood" OR "Umbilical cord blood " OR "Umbilical blood" OR Semicongruent) NEAR/3 (Grafting OR Transplantation*)) OR Halfmatched OR "Half matched" OR "allo-HSCT"):ti,ab,kw<br>#3 #1 and #2<br>#4 (Neutropenia* OR neutropenic OR neutropaenia* OR "Barth syndrome" OR "Evans syndrome"):ti,ab,kw<br>#5 (((Renal OR Kidney) NEAR/3 (Insufficienc* OR Failure* OR dysfunction*)) OR "Acute Kidney Injur*" OR "Acute Renal Injur*" OR "Lower Nephron Nephrosis" OR "Lower Nephron Nephroses" OR "Kidney Tubular Necrosis" OR "Kidney Tubular Necroses" OR "Cardio Renal |

| | |
|---|---|
| | Syndrome*" OR "Reno-Cardiac Syndrome*" OR "Renocardiac Syndrome*" OR "Cardiorenal Syndrome*" OR "Chronic Kidney Disease*" OR "Chronic Renal Disease*" OR "Frasier Syndrome*"):ti,ab,kw<br>#6 ("graft dysfunction*" OR "Poor graft function*" OR PGF OR IPGF):ti,ab,kw<br>#7 ("Re-hospital*" OR readmis* OR "re-admit*" OR readmit* OR "re-admis*"):ti,ab,kw<br>#8 MeSH descriptor: [Bacterial Infections] explode all trees<br>#9 (bacteri* OR Actinomycetal* OR Corynebacteri* OR diphtheria OR actinomycos* OR mycobacterios* OR leprosy OR paratuberculos* OR tuberculo* OR nocardios* OR anthrax OR melioidos* OR Bacteremia OR Septicemia OR Anaplasmata* OR Anaplasmos* OR Bartonella* OR Bordetella OR Brucellos* OR Burkholderia* OR Campylobact* OR Chlamydiac* OR Cytophagac* OR Desulfovibrionac* OR Ehrlichios* OR Enterobacteriac* OR Flavobacteriac* OR Fusobacteriac* OR Helicobacter* OR Legionellos* OR Moraxellac* OR Mycoplasmata* OR Neisseriac* OR Pasteurellac* OR Piscirickettsiac* OR Pseudomon* OR Rickettsiac* OR Spirochaetal* OR Treponema* OR Tularemia OR Vibrio OR Actinomycet* OR Bacillac* OR Clostridi* OR Erysipelothr* OR Listerios* OR Staphylococc* OR Streptococc* OR Chancroid OR Chlamydia OR Vaginos*):ti,ab,kw<br>#10 #4 OR #5 OR #6 OR #7 OR #8 OR #9<br>#11 #3 AND #10 |
| Web of Science | #1. TS=(Cytomegalovirus* OR "Salivary Gland Virus*" OR "Human Herpesvirus 5" OR "HHV 5" OR Cytomegalic OR CMV OR HCMV OR Cytomegaly OR cytomegalia OR cytomegalo OR cytomegalus OR cytomegalusvirus* OR "human herpes virus 5" OR cytomegaloinfection* OR cytomegaloviral)<br>#2. TS=(((Allogeneic OR allogenic OR allograft OR allotransplantation* OR Homologous OR Homograft* OR Allo) NEAR/4 ("Bone Marrow" OR "stem cell" OR "hematopoietic cell") NEAR/4 (Grafting OR Transplantation*)) OR ((Haploidentical OR "unrelated donor" OR "Cord Blood" OR "Umbilical cord blood " OR "Umbilical blood" OR Semicongruent) NEAR/3 (Grafting OR Transplantation*)) OR Halfmatched OR "Half matched" OR "allo-HSCT")<br>#3. #1 AND #2<br>#4. TS=(Neutropenia* OR neutropenic OR neutropaenia* OR "Barth syndrome" OR "Evans syndrome")<br>#5. TS=(((Renal OR Kidney) NEAR/3 (Insufficienc* OR Failure* OR dysfunction*)) OR "Acute Kidney Injur*" OR "Acute Renal Injur*" OR "Lower Nephron Nephrosis" OR "Lower Nephron Nephroses" OR "Kidney Tubular Necrosis" OR "Kidney Tubular Necroses" OR "Cardio Renal Syndrome*" OR "Reno-Cardiac Syndrome*" OR "Renocardiac Syndrome*" OR |

| | |
|---|---|
| | "Cardiorenal Syndrome*" OR "Chronic Kidney Disease*" OR "Chronic Renal Disease*" OR "Frasier Syndrome*")<br>#6. TS=("graft dysfunction*" OR "Poor graft function*" OR PGF OR IPGF)<br>#7. TS=("Re-hospital*" OR readmis* OR "re-admit*" OR readmit* OR "re-admis*")<br>#8. TS=(bacteri* OR Actinomycetal* OR Corynebacteri* OR diphtheria OR actinomycos* OR mycobacterios* OR leprosy OR paratuberculos* OR tuberculo* OR nocardios* OR anthrax OR melioidos* OR Bacteremia OR Septicemia OR Anaplasmata* OR Anaplasmos* OR Bartonella* OR Bordetella OR Brucellos* OR Burkholderia* OR Campylobact* OR Chlamydiac* OR Cytophagac* OR Desulfovibrionac* OR Ehrlichios* OR Enterobacteriac* OR Flavobacteriac* OR Fusobacteriac* OR Helicobacter* OR Legionellos* OR Moraxellac* OR Mycoplasmata* OR Neisseriac* OR Pasteurellac* OR Piscirickettsiac* OR Pseudomon* OR Rickettsiac* OR Spirochaetal* OR Treponema* OR Tularemia OR Vibrio OR Actinomycet* OR Bacillac* OR Clostridi* OR Erysipelothr* OR Listerios* OR Staphylococc* OR Streptococc* OR Chancroid OR Chlamydia OR Vaginos*)<br>#9. #3 AND (#4 OR #5 OR #6 OR #7 OR #8) |
