# Supplementary material for: The indirect effects of CMV reactivation on patients following allogeneic hematopoietic stem cell transplantation: an evidence mapping
Source: Ann Hematol. 2024 Jan 16;103(3):917–33. doi: 10.1007/s00277-023-05509-7 (PMC10866798; doi:10.1007/s00277-023-05509-7)
Supplement: Supplementary file 2 — Supplementary file2 (PDF 164 KB) [file 277_2023_5509_MOESM2_ESM.pdf]

Annals of Hematology

The Indirect Effects of CMV Infection on Patients with Allogeneic Hematopoietic Stem Cell transplantation: an Evidence Mapping

Xiaojin Wu[1,2,3], Xiao Ma[1,2], Tiemei Song[1,2], Jie Liu[4], Yi Sun [4], Depei Wu[1,2*]

1. The First Affiliated Hospital of Soochow University, Suzhou, 215000, China

2. National Clinical Research Center for Hematologic Diseases, Jiangsu Institute of Hematology, Suzhou, 215000, China.

3. Institute of Blood and Marrow Transplantation, Collaborative Innovation Center of Hematology, Soochow University, Suzhou, 215000, China.

4. MRL Global Medical Affairs, MSD China, Shanghai, 200233, China.

Corresponding author:

Depei Wu, National Clinical Research Center for Hematologic Diseases, Jiangsu Institute of Hematology, The First Affiliated Hospital of Soochow University, 188 Shizi Street, Suzhou, Jiangsu Province 215006, China. Email: drwudepei@163.com .

Online Resource 2 Quality of included systematic reviews

Item	Giménez 2019	Chuleerarux 2021	Zhang 2019
**1. Did the research questions and inclusion criteria for the review include the components of PICO?**<br>Yes/No	Yes	Yes	Yes
**2. Did the report of the review contain an explicit statement that the review methods were established prior to the conduct of the review and did the report justify any significant deviations from the protocol?**<br>Yes/Partial Yes/No	Partial Yes	Partial Yes	Partial Yes
**3. Did the review authors explain their selection of the study designs for inclusion in the review?**<br>Yes/No	Yes	Yes	No
**4. Did the review authors use a comprehensive literature search strategy?**<br>Yes/Partial Yes/No	Partial Yes	Partial Yes	Partial Yes
**5. Did the review authors perform study selection in duplicate?**<br>Yes/No	Yes	Yes	Yes
**6. Did the review authors perform data extraction in duplicate?**<br>Yes/No	Yes	No	Yes
**7. Did the review authors provide a list of excluded studies and justify the exclusions?**<br>Yes/Partial Yes/No	Partial Yes	No	No
**8. Did the review authors describe the included studies in adequate detail?**<br>Yes/Partial Yes/No	Partial Yes	Partial Yes	Partial Yes
**9. Did the review authors use a satisfactory technique for assessing the risk of bias (RoB) in individual studies that were included in the review?**<br>Yes/Partial Yes/No/Includes only NRSI/Includes only RCTs	No	Yes	Yes

**10. Did the review authors report on the sources of funding for the studies included in the review?**<br>Yes/No	No	No	No
**11. If meta-analysis was performed did the review authors use appropriate methods for statistical combination of results?**<br>Yes/No/No meta-analysis conducted	Yes	Yes	Yes
**12. If meta-analysis was performed, did the review authors assess the potential impact of RoB in individual studies on the results of the meta-analysis or other evidence synthesis?**<br>Yes/No/No meta-analysis	No	No	No
**13. Did the review authors account for RoB in individual studies when interpreting/ discussing the results of the review?**<br>Yes/No	Yes	Yes	No
**14. Did the review authors provide a satisfactory explanation for, and discussion of, any heterogeneity observed in the results of the review?**<br>Yes/No	Yes	Yes	Yes
**15. If they performed quantitative synthesis did the review authors carry out an adequate investigation of publication bias (small study bias) and discuss its likely impact on the results of the review?**<br>Yes/No/No meta-analysis conducted	Yes	Yes	Yes
**16. Did the review authors report any potential sources of conflict of interest, including any funding they received for conducting the review?**<br>Yes/No	Yes	Yes	Yes
**Overall assessment**<br>High/Moderate/Low/Critically low	Low	Low	Critically low
