# Supplementary material for: The indirect effects of CMV reactivation on patients following allogeneic hematopoietic stem cell transplantation: an evidence mapping
Source: Ann Hematol. 2024 Jan 16;103(3):917–33. doi: 10.1007/s00277-023-05509-7 (PMC10866798; doi:10.1007/s00277-023-05509-7)
Supplement: Supplementary file 3 — Supplementary file3 (PDF 167 KB) [file 277_2023_5509_MOESM3_ESM.pdf]

Annals of Hematology

The Indirect Effects of CMV Infection on Patients with Allogeneic Hematopoietic Stem Cell transplantation: an Evidence Mapping

Xiaojin Wu[1,2,3], Xiao Ma[1,2], Tiemei Song[1,2], Jie Liu[4], Yi Sun [4], Depei Wu[1,2*]

1. The First Affiliated Hospital of Soochow University, Suzhou, 215000, China

2. National Clinical Research Center for Hematologic Diseases, Jiangsu Institute of Hematology, Suzhou, 215000, China.

3. Institute of Blood and Marrow Transplantation, Collaborative Innovation Center of Hematology, Soochow University, Suzhou, 215000, China.

4. MRL Global Medical Affairs, MSD China, Shanghai, 200233, China.

Corresponding author:

Depei Wu, National Clinical Research Center for Hematologic Diseases, Jiangsu Institute of Hematology, The First Affiliated Hospital of Soochow University, 188 Shizi Street, Suzhou, Jiangsu Province 215006, China. Email: drwudepei@163.com .

Online Resource 3 Quality of included primary clinical studies

Study ID	Overall risk of bias	Study participants	Study attrition	Prognostic factor measurement	Outcome assessment	Study confounding	Statistical analysis and reporting
Chen 2022	Moderate	Moderate	Low	Low	Low	Moderate	Low
Miguel 2018	Moderate	Moderate	Moderate	Moderate	Moderate	Moderate	Moderate
Hama 2020	Moderate	Moderate	Moderate	Moderate	Moderate	Moderate	Low
Madsen 2020	Moderate	Moderate	Moderate	Moderate	Moderate	Moderate	Moderate
Levrat 2016	Moderate	Moderate	Moderate	Moderate	Moderate	Moderate	Moderate
Beswick 2018	Moderate	Moderate	Low	Moderate	Moderate	Moderate	Low
Prabahran 2021-a	Moderate	Moderate	Moderate	Moderate	Moderate	Moderate	Moderate
Saullo 2020	Moderate	Low	Moderate	Low	Low	Moderate	Low
Sun 2019-a	Moderate	Moderate	Moderate	Moderate	Low	Moderate	Moderate
Yamagishi 2018	Moderate	Moderate	Moderate	Moderate	Low	Moderate	Low
Deconinck 2005	Moderate	Moderate	Moderate	Moderate	Low	Moderate	Low
Lavallée 2016	Moderate	Low	Moderate	Low	Low	Moderate	Low
Lv 2021	Moderate	Moderate	Moderate	Moderate	Low	Moderate	Low
Prabahran 2021-b	Moderate	Moderate	Moderate	Moderate	Low	Moderate	Low
Sano 2017	Moderate	Moderate	Moderate	Low	Low	Moderate	Low
Schelfhout 2019-a	Moderate	Low	Moderate	Moderate	Low	Moderate	Moderate

Skert 2014	Moderate	Moderate	Moderate	Moderate	Low	Moderate	Low
Sun 2019-b	Moderate	Moderate	Moderate	Moderate	Low	Moderate	Low
Teschner 2022	Moderate	Low	Moderate	Moderate	Moderate	Moderate	Moderate
Vinuesa 2016	Moderate	Moderate	Moderate	Low	Low	Moderate	Low
Xiao 2014	Moderate	Moderate	Moderate	Low	Low	Moderate	Low
Lin 2022	Moderate	Low	Moderate	Low	Low	Moderate	Low
